# A unified convention for biological assemblies with helical symmetry

**DOI:** 10.1107/S0907444911024024

**Published:** 2011-07-12

**Authors:** Chung-Jung Tsai, Ruth Nussinov

**Affiliations:** a Basic Science Program, SAIC-Frederick Inc., Center for Cancer Research Nanobiology Program, NCI-Frederick, Frederick, MD 21702, USA; b Sackler Institute of Molecular Medicine, Department of Human Genetics and Molecular Medicine, Sackler School of Medicine, Tel Aviv University, Tel Aviv 69978, Israel

**Keywords:** symmetry, X-ray fibre diffraction, EM density maps, helical assemblies

## Abstract

Assemblies with helical symmetry can be conveniently formulated in many distinct ways. Here, a new convention is presented which unifies the two most commonly used helical systems for generating helical assemblies from asymmetric units determined by X-ray fibre diffraction and EM imaging. A helical assembly is viewed as being composed of identical repetitive units in a one- or two-dimensional lattice, named 1-­D and 2-D helical systems, respectively. The unification suggests that a new helical description with only four parameters [*n*_1_, *n*_2_, twist, rise], which is called the augmented 1-D helical system, can generate the complete set of helical arrangements, including coverage of helical discontinuities (seams). A unified four-parameter characterization implies similar parameters for similar assemblies, can eliminate errors in reproducing structures of helical assemblies and facilitates the generation of polymorphic ensembles from helical atomic models or EM density maps. Further, guidelines are provided for such a unique description that reflects the structural signature of an assembly, as well as rules for manipulating the helical symmetry presentation.

## Abstract 2

A new representation of helical structure by four parameters, [*n*_1_, *n*_2_, twist, rise], is able to generate an entire helical construct from asymmetric units, including cases of helical assembly with a seam.

## 1. Introduction

Under physiological conditions, many biomolecules are either organized in functional tubular forms or aggregated in disease-related filaments. Tubular and filamentous structures grow with a helical symmetry. The determination of helical structures is important because it provides clues to functional regulation and to the mechanisms of polymerization and depolymerization, and can help in figuring out how to prevent unwanted disease-related fibril aggregation. The most famous example is the determination of the Watson–Crick double-helical DNA structure in 1953 (Watson & Crick, 1953 ▶), which created a new era in the history of molecular biology. In proteins, even a slight difference in the interactions between molecules is sufficient to create similar filamentous or tubular structures with distinct helical symmetries. For this reason, structural polymorphism is a common characteristic of tubular or fibril entities. Depending on the specificity and rigidity of the interacting molecules, some, such as the amyloidogenic peptide Aβ_1–40_ (Sachse *et al.*, 2008 ▶; Schmidt *et al.*, 2009 ▶), can exhibit a broad spectrum of polymorphic assemblies, whereas others only show limited variability, as in the case of microtubules (Sui & Downing, 2010 ▶). This underscores the importance of revealing the structural characteristics of helical assemblies directly from a simple helical symmetry description.

Helical symmetry can be formulated in many different ways. Helical transformations can be classified into two categories: one-dimensional (1-D) helical systems and two-dimensional (2-D) helical systems. In structural determination by X-ray fibre diffraction (Klug *et al.*, 1958 ▶), a helical structure is described as a set of *n* 1-D molecular helices related by an *n*-­fold axial symmetry. However, in both systems, if the helically repeating motif has *C*_2_ symmetry, the helical structure has an additional dyad symmetry (Klug *et al.*, 1958 ▶). Given the asymmetric units, the helical assembly can be constructed by rotohelical transformations which are defined by a specified line group (Damnjanović *et al.*, 2007 ▶). Because of the prevalence of structural polymorphism (DeRosier *et al.*, 1999 ▶) and helical discontinuities (seams; Kikkawa, 2004 ▶) in electron-microscopy (EM) images, a description of helical assemblies by a rolled planar 2-D lattice sheet was devised to solve the EM structure in reciprocal space. In this representation, the framework of a helical structure is viewed as a helical net; that is, a set of equivalent points wrapped around a cylindrical surface. Various 2-D lattice wrappings were defined by a circumference vector **c** = *n*_1_ **a** + *n*_2_ **b**, where *n*_1_ and *n*_2_ are two integer constants and **a** and **b** are the 2-D lattice vectors. On the other hand, the reconstruction of helical structures in real space is typically based on rotohelical transformations which are applied iteratively using the single-particle method (Sachse *et al.*, 2007 ▶; Egelman, 2007 ▶, 2010 ▶).

A simple convention for defining the helical symmetry of biological assemblies has been suggested in the remediated Protein Data Bank (PDB; Lawson *et al.*, 2008 ▶) and EM Data Bank (Heymann *et al.*, 2005 ▶) archives. Both have used a definition of rotohelical transformation that does not fully capture the underlying symmetry properties of helical assemblies. It is therefore not surprising that two very similar tubular structures might be described by very different helical parameters that provide no clue to the fact that they are actually quite similar. This is the case for the two bacterio­phage major coat protein helical tubes determined by X-ray fibre diffraction [PDB entries 1hgv (Pederson *et al.*, 2001 ▶) and 1ifd (Marvin, 1990 ▶)]; the first is presented as a one-start and the second as a five-start helical tube with each helix related by a fivefold rotational symmetry. The discrepancy is understandable because the two PDB structures were the outcome of structure-determination procedures in which the helical symmetry was preset in the minimization procedure. This shortcoming underscores the importance of a standard system that would report helical structures and provide parameters that reflect their structural characteristics. It appears that to date an unambiguous, simple and systematic standard for defining a unique helical specification for constructing helical assemblies from asymmetric units is lacking.

In this paper, we present a new unified convention for the construction of helical assemblies from asymmetric units determined by X-ray fibre diffraction and EM imaging. The unification is made possible by an augmented 1-D helical system (described below) that extends the traditional 1-D helical scheme to adopt the helical symmetry descriptor [*n*_1_, *n*_2_] which is used in the 2-D helical system. A helical structure can be prepared by rolling a planar sheet composed of identical 2-D unit cells (Stewart, 1988 ▶). In order to create a seamless 2-D lattice tube, two integer constants [*n*_1_, *n*_2_] define the wrapping process: *n*_1_ refers to the number of cells that are needed to complete a full round of cylinder wrapping and *n*_2_ to the number of cells sliding along the cell edge after the wrapping. The helical symmetry of the tubular structure is explicitly determined by [*n*_1_, *n*_2_] and the corresponding 2-D wrapping transformations can be found in the literature (Tsai *et al.*, 2006 ▶; Kikkawa, 2004 ▶).

In a traditional 1-D helical system, a helical structure is depicted as either a one-start or an *n*-start helical structure (Egelman, 2007 ▶; Klug *et al.*, 1958 ▶). For a one-start helical structure, the assembly consists of only a single helix with two helical parameters, twist (ϕ) and rise (δ); these denote the transformation of the 1-D unit cell which is used to build the entire structure. In fibre diffraction, a one-start helix is formulated as *u* units in *v* turns with a helical repeat distance of *c*, which straightforwardly gives ϕ = 2π*v*/*u* and δ = *c*/(*uv*). An *n*-start helical structure has *n* helices related by an *n*-fold axial symmetry (*C_n_*), with the axis coinciding with the helical axis. In the augmented 1-D helical system described below, in addition to the rotational operation of the *C_n_* symmetry there is an extra translational operation along the helical axis. In the 2-D helical system this extra translational operation is implicitly included in the 2-D wrapping transformation; however, it is ignored in the traditional 1-D helical scheme. This prevents the 1-D and 2-D systems from being unified in a common helical symmetry description. In contrast, the helical symmetry in the augmented 1-D helical system with the four parameters [*n*_1_, *n*_2_, twist, rise] is defined by two consecutive helical (screw) operations: the first helical operation is specified by two helical parameters [twist, rise] exactly as in the traditional 1-D helical transformation and the second screw operation is defined by two [*n*_1_, *n*_2_] constants. *n*_1_ refers to the *n*_1_-fold rotational symmetry exactly as in the traditional 1-D helical transformation and *n*_2_ specifies the translation part of the second screw operation. We will illustrate the operational transformations as well as the interconversion between the augmented 1-D helical system and the 2-D helical system below.

## 2. Methods

In our definition, a helical structure is composed of *repetitive identical* units, similar to a single crystal which is built from a three-dimensional (3-D) lattice. The definition of repetitive relates to the entire helical structures, which are built from a unit cell with a specified helical symmetry. The unit cell is either a 2-D lattice or a 1-D line segment. The definition of identical implies that each repetitive unit in the construct has exactly the same environment; that is, the independent variables of a helical structure include only parameters involved in helical transformation and coordinates of asymmetric units within a unit cell. Identical implies that when evaluating the energy of the assembly there is no need to include the interactions between all units but just the interactions between one unit cell and its surrounding cells.

Given a 3-D unit cell, it is straightforward to generate the entire single crystal with fractional coordinates. The newly generated fractional coordinates are the number of cells (*n_a_, n_b_, n_c_*) away from the origin (0, 0, 0) along each edge. The Cartesian coordinates of a new cell can be converted from the fractional coordinates by an orthogonal matrix (Evans, 2001 ▶) computed from 3-D lattice constants, *a*, *b*, *c*, α, β and γ. However, unlike the 3-D crystal system, a specified helical transformation is needed to generate the helical assembly from a given 1-D or 2-D unit cell. In the following, the equations for the 2-D wrapping and the augmented 1-D helical transformation will be derived and explained in detail.

### 2.1. 2-D helical system

As stated in §[Sec sec1]1, a planar sheet of 2-D lattice can be wrapped into a tube. If all sheet units are identical, the two constant integers *n*_1_ and *n*_2_ are sufficient to define all possible distinct tubes obtained by rolling it. Here, *n*_1_ is the number of cells along one edge of the 2-D lattice (*a*) which are required to make a full round of wrapping and *n*_2_ refers to the number of cells sliding along the other edge of the lattice (*b*) after wrapping.

**Figure 1:**
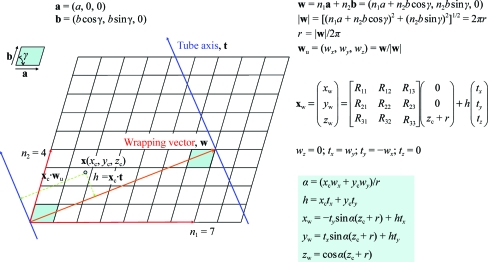
A brief graphic summary of the 2-D helical system. On the left, a 2-D lattice wrapping specified by a circumference vector, **w** = *n*_1_ **a** + *n*_2_ **b**, is sketched with an example of *n*_1_ = 7 and *n*_2_ = 4. The 2-D lattice is highlighted in color with the angle γ between the two axes **a** and **b**. The lattice lies on the Cartesian *xy* plane and the axis **a** lies along the Cartesian *x* axis. Thus, the axis **a** in Cartesian coordinates is (*a*, 0 ,0) and **b** is (*b*cosγ, *b*sinγ, 0). The wrapped helical coordinates (*x*_w_, *y*_w_, *z*_w_) of the Cartesian coordinates (*x*_c_, *y*_c_, *z*_c_) in the 2-D planar system can then be calculated by the helical transformation in terms of the helical axis **t** (which is perpendicular to **w**) with the two parameters α and *h*, twist angle and rise distance, respectively. It is then straightforward to determine the circumferential unit vector **w**_u_, the helical radius *r* and the helical axis **t** as formulated on the right-hand side of the sketch with the summarized wrapping equations highlighted in color at the bottom. Note that vector (*t_x_*, *t_y_*, *t_z_*) as calculated from **w**_u_ is also a unit vector.

If we place edge **a** along the *x* axis of the Cartesian co­ordinate and a 2-D lattice (*a*, *b*, γ) is placed on the *xy* plane, the wrapping equations for an [*n*_1_, *n*_2_] tube are
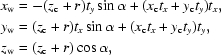
where
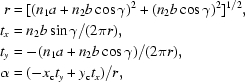
(*x*_w_, *y*_w_, *z*_w_) are the wrapped Cartesian coordinates of the tube and (*x*_c_, *y*_c_, *z*_c_) are the Cartesian coordinates of the associated 2-D sheet. In the wrapping equations, the 2-D lattice sheet is at a distance of the tube radius (*r*) from the tube axis (*t_x_*, *t_y_*, 0). The helical transformation is implicitly specified by the helical twist α and the helical rise *x*_c_ *t_x_* + *y*_c_ *t_y_*. Fig. 1 ▶ provides a graphical summary of the 2-D helical transformation. A more detailed description has been given previously (Tsai *et al.*, 2006 ▶). Given asymmetric units in a 2-D lattice and a helical symmetry specified by the 2-D helical system in five parameters [*n*_1_, *n*_2_, *a*, *b*, γ], one can build a complete helical construct based on the 2-D helical transformation equations formulated above.

### 2.2. Augmented 1-D helical system

Instead of rolling a planar 2-D sheet, a helical structure can also be expressed by a single helix or *n* helices, with the *n* helices related by an *n*-fold screw axis instead of just a rotational axis. Because the helical assembly must consist of identical subunits, the rotational part of the screw axis must display a *C_n_* rotational symmetry and the translational part should be limited by some discrete numbers. In the augmented 1-D helical system, the four parameters [*n*_1_, *n*_2_, ϕ, δ] indicate that there are *n*_1_ helices in the assembly, with each individual helix characterized by a unit twist (ϕ) and a unit rise (δ). Because the helices are also related by an *n*_1_-fold screw axis, each helix denoted by *m*_1_ = 0, 1, 2, …, *n*_1_ − 1 has an additional twist of *m*_1_(2π/*n*_1_) and a rise of *m*_1_(*n*_2_/*n*_1_)δ. Note that the rise, which is specified by *n*_2_ with a quantity of *n*_2_/*n*_1_δ, was not included in the traditional 1-D helical system. Note also that *m*_1_ = *n*_1_ refers back to the first helix as specified by (ϕ, δ), which will give *n*_2_δ rise after a complete round of *n*_1_ rotations. In the 2-­D helical system, this corresponds to the number of cells involved in the helix sliding after a complete wrapping.

**Figure 2:**
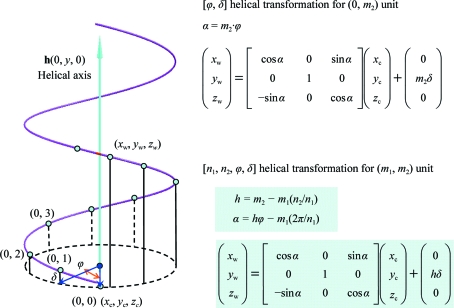
A brief graphic summary of the 1-D helical system. For simplicity, on the left-hand side of the figure, only a single helix is drawn to illustrate the 1-D helical transformation. The helical axis **t** is along the Cartesian *y* axis and the 1-D cell (asymmetric subunits) is sitting at (*x*_c_, *y*_c_, *z*_c_) which is labeled as the (0, 0) cell. The transformed Cartesian coordinates (*x*_w_, *y*_w_, *z*_w_) labeled as (0, *m*_2_) accordingly are calculated by the matrix operations with a rotational angle of *m*_2_ϕ and a translational rise of *m*_2_δ in the upper right-hand side of the figure. For the augmented 1-D helical system with four parameters [*n*_1_, *n*_2_, ϕ, δ], the helical transformation equation expressed by matrix operations is given in the lower right corner highlighted in color. α and *h*δ respectively specify the overall helical twist and helical rise for the (*m*_1_, *m*_2_) subunits with respect to the (0, 0) asymmetric subunits, with *m*_1_ referring to the *n*_1_-order helix and *m*_2_ referring to the subunits along the denoted helix.

A helical structure in the symmetrical construct is identified by the cell coordinates [*m*_1_, *m*_2_]. The asymmetric units are given in cell [0, 0] and an [*m*_1_, *m*_2_] cell is located in the *m*_1_ helix *m*_2_ units away from the cell [*m*_1_, 0] along the helix. If the helical axis is parallel to the *y* axis and passes through the origin (0, 0, 0), the helical transformation equations for an [*m*_1_, *m*_2_] cell in an [*n*_1_, *n*_2_] helical construct are
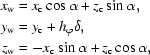
where
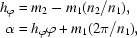
(*x*_w_, *y*_w_, *z*_w_) are the transformed Cartesian coordinates for the cell [*m*_1_, *m*_2_] and (*x*_c_, *y*_c_, *z*_c_) are the Cartesian coordinates of asymmetric units in cell [0, 0]. *h*_ϕ_ and α specify the overall rise and twist for the [*m*_1_, *m*_2_] cell as specified by an *n*_1_-fold screw axis with *n*_2_ unit shift. In the case of a helical structure with a single helix, in which *n*_1_ = 1 and *m*_1_ = 0, the helical transformation above reduces to a simple helical operation defined by [ϕ, δ] only. A graphical summary of the augmented 1-D helical transformation is given in Fig. 2 ▶.

### 2.3. 2-D helical system → augmented 1-D helical system

There are four ways to convert a helical system from 2-D to 1-D: view the continuation of lattice edge **b** as a helix, view the continuation of lattice edge **a** as a helix or view the continuation along the vector of **a** + **b** or along the vector of **a** − **b**. The first is the most convenient choice. By selecting the vector **b** as an individual helix, the new 1-D helical system retains the same symmetry notation as the 2-D helical system [*n*_1_, *n*_2_]. The unit twist ϕ (in unit of radians) and rise δ of the *n*_1_-­start helices are calculated as
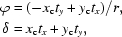
where *t_x_*, *t_y_* are the helical axes of the 2-D helical system and *x*_c_, *y*_c_ are the planar Cartesian coordinates at the cell origin (0, 1). Because the tube axis of the 1-D helical tube (along the *y* axis) is different from the 2-D helical tube (on the *xy* plane), the Cartesian coordinates referenced in the 2-D system require some transformations in order to correspond to the new 1-D system. This can be performed either directly in wrapped Cartesian coordinates or in native fractional co­ordinates. In Cartesian coordinates, the transformation is equivalent to aligning the 2-D helical axis of the *xy* plane back onto the *y* axis with the *z* axis (0, 0, 1) as the rotational axis. Under the right-handed rotational system, the angle θ between the old tube axis and the *y* axis is calculated as atan(−*t_x_*/*t_y_*) and the transformations are
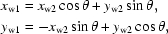
where *x*_w1_, *y*_w1_ are the wrapped Cartesian coordinates of the new 1-D helical system and *x*_w2_, *y*_w2_ are the wrapped Cartesian coordinates of the old 2-D helical system.

### 2.4. Augmented 1-D helical system → 2-D helical system

The conversion from a 1-D to a 2-D helical system is not as straightforward as the opposite conversion. This is partly because a 2-D lattice is loosely defined by a single helix structure and partly because of the necessity to revert from the wrapped tube coordinates back to 2-D planar Cartesian coordinates. Given a 1-D helical structure, we first calculate the implicit helical radius from the center of mass of the representative units by assuming that the center of the 1-D helical assembly is located at the origin (0, 0, 0). Secondly, we either use the original *n*_1_ of the 1-D system or determine a new *n*_1_ for the 2-­D helical system and define accordingly two wrapped coordinates, (*x*_w1_, *y*_w1_, *z*_w1_) and (*x*_w2_, *y*_w2_, *z*_w2_), from the 1-D helical system to serve as the origin of cells (1, 0) and (0, 1) of the new 2-D helical system, respectively. Thirdly, we reverse the two wrapped coordinates back to unwrapped planar Cartesian coordinates, (*x*_c1_, *y*_c1_, *z*_c1_) and (*x*_c2_, *y*_c2_, *z*_c2_). With the new calculated planar coordinates, it is straightforward to calculate the 2-D lattice constants as follows:
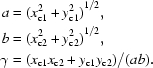
Fourthly, given the newly determined *r*, *a*, *b*, γ and *n*_1_, *n*_2_, the new 2-D helical tube can be determined by solving the quadratic equation *b* ^2^ *x* ^2^ + 2*n*_1_ *ab* cos γ *x* + (*n*_1_ *a*)^2^ − (2π*r*)^2^ = 0. Finally, all Cartesian coordinates of the old 1-D system are reversed back to the new 2-D planar coordinates.

### 2.5. Properties of [*n*_1_, *n*_2_] helical system

**Figure 3:**
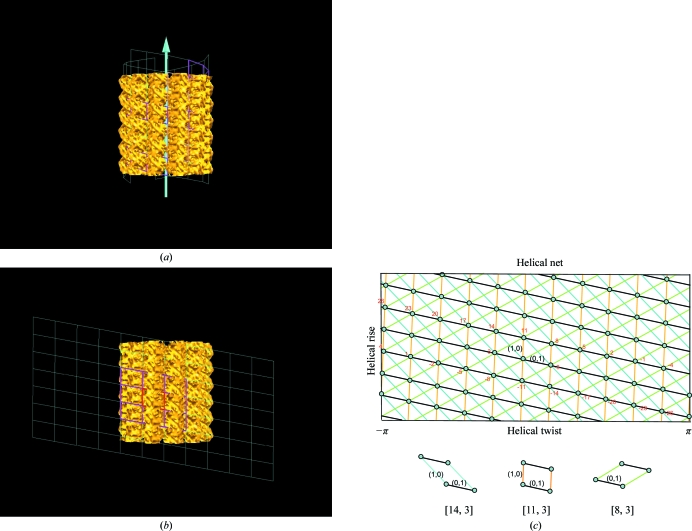
Illustrations of [*n*_1_, *n*_2_] helical symmetry with respect to helical nets. In (*a*), an EM segment of the microtubule structure is shown in a wrapped helical net with [11, 3] symmetry. The corresponding flattened unwrapped helical net is demonstrated in (*b*). A section of the corresponding [11, 3] helical net is drawn in (*c*), with the *x* axis covering the helical circumference, a twist range of 2π and the *y* axis parallel to the helical axis, corresponding to the helical rise. The colored circular dots in the net are the asymmetric subunits and a solid line that passes through a set of dots is a helix. A helical net can be redefined by any two sets of lines with their intersections covering all dots. With *n*_2_ fixed at 3, there are ten additional sets of helices which can be used to define the same helical structure. See text for an explanation of why only limited sets of helices are feasible with *n*_2_ fixed at 3. In (*c*), feasible sets of helices are marked beside the dots with the value of *n*_1_ colored red. Two of the new helical nets with helical symmetry [8, 3] and [14, 3] are superimposed in (*c*). The individual lattice drawn under the helical nets is to help in the visualization of individual helical nets.

We have shown that both the 1-D and 2-D helical systems can be represented by two integers, [*n*_1_, *n*_2_], and that the helical assembly can be built through the helical transformation with the associated parameters. However, the helical symmetry specified by these two integers can also be interpreted in a way different from the helical systems’ definitions. In the traditional helical description, an assembly with [*n*_1_, *n*_2_] symmetry can be viewed as two sets of *n*-start helices in which either the arrangement of the *n*_1_-start helices is specified by *n*_2_ or, *vice versa*, that of the *n*_2_-start helices is specified by *n*_1_. The best way to illustrate [*n*_1_, *n*_2_] helical symmetry is by using a helical net: an unwrapped flattened 2-D net bound by the circumference in one direction and extended to infinity parallel to the helical axis. Figs. 3 ▶(*a*) and 3 ▶(*b*) illustrate an example of wrapping and unwrapping of the helical net with the EM structure of a microtubule with [11, 3] symmetry (Sui & Downing, 2010 ▶). The colored circular dots in the helical net represent asymmetric units and a line passing through a set of dots is a helix. The number of intersections (*n*) between the set of parallel lines with the circumference is exactly the number *n* of helices that are required to fill the helical assembly. This is the origin of the *n*-start helices definition. In terms of a helical net description, the helical symmetry can be specified by picking a particular set of two intersecting lines (helices) corresponding to *n*_1_-start and *n*_2_-start lines. The intersections define the locations of repeating asymmetric units in the helical structure. In addition to the [11, 3] symmetry, two feasible helical nets with symmetries [8, 3] and [14, 3] are also depicted in Fig. 3(*c*) for the same helical structure. In the special case of a one-start helix, the entire assembly is built from a single helix instead of a set of helices (*n*-start helices). Although *n*_2_ is not required in helical symmetry denoted as a one-start helix, it is still represented in the [1, *n*_2_] notation.

Based on the augmented 1-D helical system, it is not difficult to realise that a helical symmetry with [*n*_1_, *n*_2_, twist, rise] is equivalent to [−*n*_1_, −*n*_2_, twist, rise], [−*n*_1_, *n*_2_, −twist, −rise] and [*n*_1_, −*n*_2_, −twist, −rise]. To reduce the redundancy in the helical symmetry representation, we set several simple rules. Firstly, *n*_1_ is always positive. For consistency in the interconversion between the 1-D and 2-D helical systems (with the sign of *n*_2_ kept unchanged), we choose the rise to also be positive. Secondly, the value of *n*_2_ is always smaller than that of *n*_1_. In this way, the handedness of the *n*_1_ helices is determined by the sign of twist: if positive the *n*_1_ helix is right-handed, otherwise it is left-handed. The sign of *n*_2_ gives the handedness of the *n*_2_ helices: if negative it is a right-handed helix, otherwise it is left-handed. To calculate the [twist, rise] of the *n*_2_-start helices the helical symmetry can be swapped from [*n*_1_, *n*_2_] to [*n*_2_, *n*_1_].

### 2.6. Local *C*_2_ (dyad) symmetry

Above, the helical symmetry operation has been applied to asymmetric subunits without first assigning a plausible local symmetry. In order to generate all symmetric subunits in a planar 2-D lattice, the local symmetry can be specified by one of the 17 wallpaper groups. However, the local symmetry in the planar 2-D lattice is largely lost by the [*n*_1_, *n*_2_] helical transformation; therefore, we only describe pseudo-local symmetry. A local *C*_2_ symmetry operation is an exception: not only is it maintained between asymmetric subunits within the unit cell, but also in the entire helical construct. In terms of the 1-D helical system, the *C*_2_ symmetry is defined as an additional dyad symmetry (with axial *C*_2_ along the *z* axis and the helical axis along the *y* axis). To include a local *C*_2_ symmetry operation, we can assign a wallpaper group *p*2 before applying the helical transformation.

### 2.7. Manipulation of [*n*_1_, *n*_2_] symmetry

A helical symmetry can be described by many [*n*_1_, *n*_2_] combinations. For a particular preset *n*_2_ there are a limited number of *n*_1_; similarly, a preset *n*_1_ will have a limited selection of *n*_2_ for the same helical assembly. To understand the manipulation and limitations of changing from one [*n*_1_, *n*_2_] to another, the helical net is the best reference. For illustration, we use the [11, 3] helical symmetry of the polymorphic helical structure of the microtubule. In terms of the (*h*, *k*; *n*) notation (Toyoshima & Unwin, 1990 ▶; Toyoshima, 2000 ▶), the set of *n*-­start helices can be specified by the equation
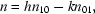
where *n*_10_ = 11 and *n*_01_ = 3 for the [11, 3] symmetry. Note that the (*h*, *k*) index has to be confined within the circumference range. Starting with the [11, 3] symmetry and a fixed *n*_2_ = 3, the redundant helical symmetry [*n*_1_, 3] can have *n*_1_ = *h* 11 − *k* 3, with *h* = ±1. On the other hand, given a fixed *n*_1_ = 11, we can have many redundant [11, *n*_2_] helical symmetries with *n*_2_ = *h* 11 − *k* 3 and *k* = ±1. In the case of (*h* = 1, *k* = ±1), we have new redundant helical symmetries of [8, 3] and [14, 3] (Fig. 3 ▶ *c*), respectively. The complete list of redundant [*n*_1_, 3] symmetries with *h* = 1 are [26, 3], [23, 3], [20, 3], [17,3], [14,3], [11,3], [8, 3], [5, 3], [2, 3], [−1, 3] and [−4, 3]. For [*n*_1_, 3] there is an infinite number of helical symmetries in a planar 2-D lattice rather than just the 11 sets restricted by the circumference. In Fig. 3 ▶(*c*), the corresponding redundant sets of helical symmetries are noted next to the helical dots. For the redundant symmetry [−1, 3], we have created an equivalence between 11-start helices [11, 3] and one-start helix [−1, 3] (or [1, −3]) symmetry.

In order to check whether the (*h*, *k*) index is within the circumference, we convert the 1-D system to its equivalent planar 2-D helical net. It is then straightforward to calculate the new helical parameters ϕ for the new *n*-start helix. If |ϕ| is less than π then it falls within the circumference range.

### 2.8. Relevance to X-ray fibre diffraction and the EM method

**Figure 4:**
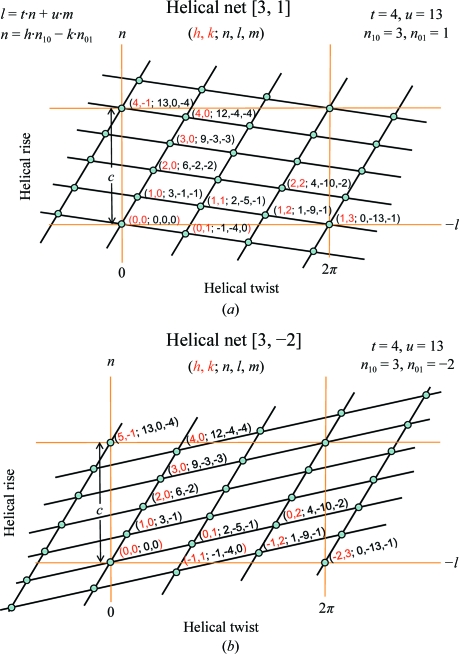
The relationship between the [*n*_1_, *n*_2_] helical scheme and the helical symmetry utilized in two common indexing systems. The dots in the figure represent two properties. Firstly, they represent asymmetric units based on a simple helical structure which is described by a one-start helix (*t* = 4 and *u* = 13) with 13 subunits and four turns completing a repeat of distance *c*. Secondly, they describe a simplified diffraction pattern of the same helical structure. Thus, instead of showing the layer-line pattern for *n*-order Bessel diffraction, each dot gives the position of (*n*, *l*) diffraction where the layer-line pattern has a maximum diffraction peak at the ∼*n* + 2 position (Diaz *et al.*, 2010 ▶). The diffraction pattern in terms of −*l* and *n* is related to the helical net description with 13 subunits enclosed by orange lines as a repeating unit. The two implicit helical symmetries, *l* = *tn* + *um* and *n* = *h n*_10_ − *k n*_01_, are then related by the [3, 1] and [3, −2] helical symmetry in (*a*) and (*b*), respectively, with *n*_10_ = *n*_1_ and *n*_01_ = *n*_2_. In the figure, each dot is labeled with an (*h*, *k*; *n*, *l*, *m*) index and the helical lines are in terms of the [*n*_1_, *n*_2_] helical symmetry.

In helical structure determination by X-ray fibre diffraction or EM based on the Fourier–Bessel method (in reciprocal space), the first step is indexing the layer-line diffraction pattern to a specified helical symmetry. There are two possible systems for indexing a diffraction pattern. In the first, assuming that a helical assembly can be described by a single (one-start) helix, the ‘selection rule’ *l* = *tn* + *um* can be utilized to assign (*n*, *l*) pairs to layer-lines in which each layer-line is associated with a set of *n*-start helices. A more general formalism using (*n*, *Z_l_*) instead of (*n*, *l*), which removes the requirement for *t*/*u* to be a rational number, is more appropriate for fibre diffraction. However, for simplicity, we prefer to use the (*n*, *l*) system here. A successful layer-line indexing then gives the helical organization as the selection rule implies: *u* units require *t* turns of the one-start helix to complete a true repeat with a rise distance of *c*. A second more systematic (*h*, *k*; *n*) indexing system (Toyoshima & Unwin, 1990 ▶; Toyoshima, 2000 ▶) interprets diffraction patterns based on the helical surface lattice. In a planar 2-D lattice, diffraction by a set of lines gives a row of dots in reciprocal space. Therefore, it is straightforward to determine a 2-D planar symmetry from an ideal diffraction pattern. For a helical structure which is obtained by wrapping of a 2-D lattice, a set of lines now becomes a set of helices and the corresponding diffraction dots become layer-lines. To define a surface lattice, two indices, (1, 0; *n*_10_) and (0, 1; *n*_01_), are first assigned where *n* is the start number of the associated helices, which can be estimated from its peak position in the layer-line diffraction (Toyoshima & Unwin, 1990 ▶; Toyoshima, 2000 ▶). If the remaining layer-lines can be indexed and related by the equation *n* = *hn*_10_ − *kn*_01_ then the helical symmetry is determined. Fig. 4 ▶ illustrates the relationship between the new [*n*_1_, *n*_2_] helical scheme and the symmetry in both indexing systems. The nodes in Fig. 4 ▶ represent (i) asymmetric units based on a simple helical structure which is described by a one-start helix (*t* = 4 and *u* = 13), *i.e.* with 13 units and four turns completing a true repeat of distance 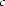, and (ii) a simplified diffraction pattern of the same helical structure. However, instead of the layer-line pattern for *n*-order Bessel diffraction, each dot gives the position of (*n*, *l*) diffraction where the layer-line pattern has a maximum diffraction peak at the ∼*n* + 2 position (Diaz *et al.*, 2010 ▶). In the figure, [*n*_1_, *n*_2_] of the new helical scheme correspond to the choice of *n*_10_ and *n*_01_ in the (*h*, *k*; *n*) indexing system. The same (*t* = 4 and *u* = 13) structure is related by two different helical systems [3, 1] and [3, −2] in Figs. 4 ▶(*a*) and 4 ▶(*b*), respectively, for a simplified diffraction pattern in terms of *n* and −*l*. The figure only shows one fourth of the diffraction pattern. For example, the *n* = 4, *l* = 3 diffraction is at the left-upper corner of the figure without a label of (*h*, *k*; *n*, *l*, *m*).

### 2.9. General guidelines for presenting a helical structure

There is no clear-cut advantage in treating a helical assembly as a 1-D or a 2-D system; both systems have pluses and minuses. However, we believe that the augmented 1-D helical system is more suitable than the 2-D system for describing a helical structure, even though the two systems are equivalent and interchangeable. There are two reasons for favoring the 1-­D helical system for describing a helical symmetry. Firstly, the 1-D helical system is simpler than the 2-­D system, with one fewer parameter. Secondly, the 1-D helical scheme is independent of the helical radius, while the surface lattice parameters (*a*, *b*, γ) in the 2-D system will change with different radii. Here, based on a 1-D helical system we suggest general guidelines for helical structure representation. With our guidelines, if the assembly units can be unambiguously defined and follow the helical paths, each helical assembly is expected to provide a unique symmetry [*n*_1_, *n*_2_, ϕ, δ] that also explicitly reflects the helical structural characteristics.

In terms of a helical net, a helical structure is composed of a set of *n* helices in which the individual helix is named an *n*-­start helix. If a helical structure can be expressed by just a single helix, it is a one-start helical structure. In the augmented 1-D helical system, the [*n*_1_, *n*_2_] representation implies that the organization of the *n*_1_ helices is specified by *n*_2_, with the individual *n*_1_-start helix defined by [ϕ, δ]. We can also swap the representation to say that there are *n*_2_ helices in the structure related by *n*_1_. Since there are many [*n*_1_, *n*_2_] combinations for a particular helical structure, the first and the most important guideline is to define the rule for choosing a unique [*n*_1_, *n*_2_] specification. In order to reflect the helical structural characteristics, the rule states that only protofilaments will be candidates for the [*n*_1_, *n*_2_] selection. In our definition, if adjacent asymmetric subunits in an assigned helix are in physical contact, this helix is a protofilament. Therefore, we first sort protofilaments according to the extent of contacts between adjacent asymmetric subunits. Of the best four protofilaments, the one with the twist angle closest to zero is set as the primary protofilament *n*_1_ and the next best protofilament is selected as the secondary protofilament *n*_2_. Note that *n*_1_ is always larger than |*n*_2_| under this guideline.

To ensure a unique helical symmetry representation for a helical assembly, redundancy needs to be reduced to singular [*n*_1_, *n*_2_, ϕ, δ]. The reduction guideline requires that *n*_1_, δ > 0 and *n*_1_ > |*n*_2_|. In the case of *n*_1_ < 0, one can apply the equivalent rule that the new [*n*_1_, *n*_2_] = [−*n*_1_, −*n*_2_]. If δ is negative, one can simply apply the equivalent rule [*n*_1_, *n*_2_, ϕ, δ] = [*n*_1_, −*n*_2_, −ϕ, −δ] to make it a positive value.

## 3. Results

There are many helical filaments and tubular structures in the PDB which have been solved either directly by X-ray fibre diffraction or by fitting individual crystal structures into cryo-EM density maps. Similar to X-ray crystal structures where only the coordinates of the asymmetric units are included in the PDB file, most of the helical structures deposited in the PDB also contain only asymmetric units. Therefore, in principle, the entire helical structures should be constructed from the deposited asymmetric units by a specified helical symmetry. In the case of crystal structures, a space group and six lattice constants are defined in the keyword ‘CRYST1’ in the PDB for calculating all symmetric units in the unit cell. However, owing to the lack of a simple, complete and widely accepted system for helical symmetry, no keyword has been set to define the helical symmetry and helical parameters are implicitly stated in the comments. Furthermore, the creation of the entire helical structure relies on a set of translational and rotational matrices which are hard-coded in the PDB.

We have applied the augmented 1-D helical scheme along with the suggested guidelines to all helical structures deposited in the PDB. A small portion of the results are given in Table 1 ▶ and a complete list is available on the web at http://protein3d.ncifcrf.gov/helicalSymmetry/table1.html. The newly determined helical parameters [*n*_1_, *n*_2_, twist, rise] not only directly reflect the helical characteristics but also provide sufficient information for constructing an entire helical structure from given asymmetric units. Here, we propose four helical parameters in a new keyword named HELSYM in the PDB for the specified helical symmetry to avoid using matrices and comments when specifying a helical symmetry.

In the PDB, the axial symmetry of a helical structure is conventionally along the *z* axis and passes through the origin (0, 0, 0). The helical symmetry specified in the PDB usually follows the rotohelical description, which provides the helical parameters (ϕ, δ) for a single helix or *n* helices related by a *C_n_* rotational axis. The manually extracted data, the helical twist ϕ and the helical rise δ, are first verified against the helical transformation matrices if also given in the PDB file. The corresponding augmented 1-D helical parameters will then be either [1, 0, ϕ, δ] or [*n*, 0, ϕ, δ] for one-start helices or *n*-start helices, respectively. We then use our graphics tool named *PNAS* (*Protein Nanoscale Architecture by Symmetry*), in-house software running both under Linux and Windows, to search for the first four protofilaments with the largest contact between the asymmetric units and determine their *n*_1_, *n*_2_, ϕ, δ values accordingly. Next, we select among them the helical protofilament with the lowest absolute value of twist angle as the primary *n*_1_ helix and its associated twist and rise are set as the 1-D helical parameters [ϕ, δ]. Finally, the highest contact protofilament other than the chosen primary helix is assigned as the secondary *n*_2_ helix to complete the determination of [*n*_1_, *n*_2_, ϕ, δ].

**Figure 5:**
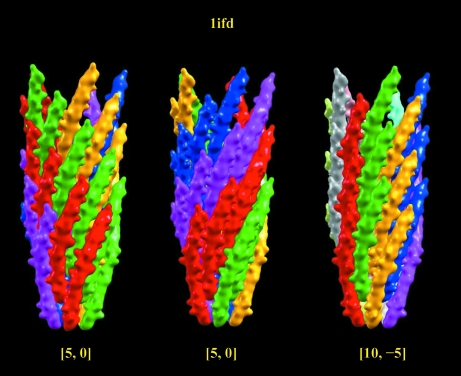
A helical structure with various distinct descriptions of helical assembly. The helical structure of bacteriophage major coat protein (PDB entry 1ifd) is used as an example here to illustrate the variations. In the figure, an individual asymmetric subunit is presented by a color isosurface entity and each color represents a helix specified by the helical symmetry. The first [5, 0] helical symmetry given in the PDB file has a corresponding 1-D helical symmetry of [5, 0, −33.2, 16.0]. The other two helical symmetry assignments, which are more meaningful in terms of reflecting structural characteristics of the helical assembly, are given as [5, 0, 38.8, 16.0] and [10, −5, 5.5, 32.0]

**Figure 6:**
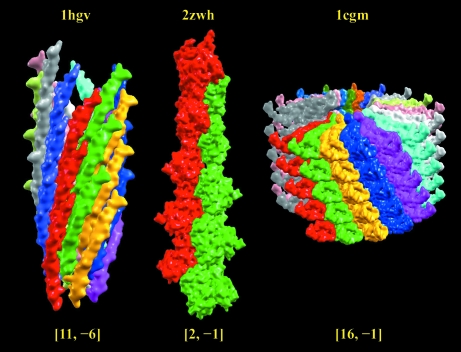
Pictorial 1-D helical symmetry description of three helical structures determined by X-ray fibre diffraction. The three depicted helical structures are the filamentous bacteriophage ph75 (PDB entry 1hgv), F-actin (PDB entry 2zwh) and the cucumber green mottle mosaic virus (PDB entry 1cgm). The same isosurface and color definition described in Fig. 5 ▶ is used for the three helical assemblies. The pictorial presentation directly indicates the helical signatures of the three helical structures in 11, two and 16 colored protofilaments, which are respectively implied by the specified [11, −5], [2, −1] and [16, −1] helical symmetry.

In Fig. 5 ▶, the helical assembly of the bacteriophage major coat protein (PDB entry 1ifd; Marvin, 1990 ▶) is assigned into three different 1-D helical symmetries: [5, 0, −33.2, 16.0], [5, 0, 38.8, 16.0] and [10, −5, 5.5, 32.0]. The first symmetry [5, 0] corresponds to the rotohelical assignment in the PDB. Apparently, each individual helix is not a protofilament since no contact between helical subunits (shown in the same color) is observed. Therefore, the helical structural characteristics will not be conveyed clearly from its helical parameters. The second [5 ,0] symmetry is based on the first protofilament with the largest number of contacts between helical subunits. However, the guideline suggests using the [10, −5] helical symmetry to represent this structure. This symmetry is advantageous for three reasons: firstly, the [10, −5] symmetry corresponding to the second and first protofilaments in the structure presents the best structural characteristics, unlike the second [5, 0] assignment which only contains information for the first protofilament; secondly, by looking down the helical axis the structure is composed of ten helices, not just five; and thirdly, another inovirus coat protein (PDB entry 1hgv; Pederson *et al.*, 2001 ▶) also gives a similar structure with [11, −6] helical symmetry. Here, the primary (11-start) helix is the first protofilament and the secondary (six-start) helix is the second protofilament. These two examples show that the augmented 1-D helical representation not only describes similar helical structures by similar parameters but at the same time also differentiates between similar helical organizations. In Fig. 6 ▶, three additional helical structures are depicted in 1-D symmetry. Pictorial descriptions with 1-D helical symmetry for the complete list of known helical structures can be accessed from links on the webpage http://protein3d.ncifcrf.gov/helicalSymmetry/table1.html.

For helical structures deposited in the EMDB (Lawson *et al.*, 2011 ▶) only the helical classification is indicated but no helical symmetry is explicitly given in the data bank. However, it is not difficult to deduce the 1-D helical parameters if the helical axis can be determined from the EM density map. The graphics tool *PNAS* can be utilized to assign 1-D helical symmetry to the EM structure. Firstly, we determine the location of the primary protofilament by visual inspection of the density map when shown in various isosurface presentations. Looking down the EM map along the helical axis, the number of assigned protofilaments can be counted to give the *n*_1_ helical parameter. *PNAS* then determines the position of the helical axis using either the given map center or the calculated coordinates of the center of density. Next, we determine the [ϕ, δ] pair for the visually assigned primary protofilament by calculating the correlation coefficient (Grubisic *et al.*, 2010 ▶) between the origin map density and the helical transformed density specified by a pair of manually adjustable parameters [ϕ, δ]. In this procedure, we follow the guideline to keep the helical twist as close to zero as possible and at the same time change the twist and rise to reach the best match as guided by visual superimposition between the origin and the transformed density map in isosurface presentation. The optimal correlation coefficient should be very close to 1.0. Now, we can use the newly determined helical parameters *n*_1_, ϕ and δ to determine *n*_2_: simply try integer numbers between −*n*_1_ and *n*_1_ and perform the 1-D helical transformation to determine *n*_2_ from the result of the superimposition as stated above.

**Figure 7:**
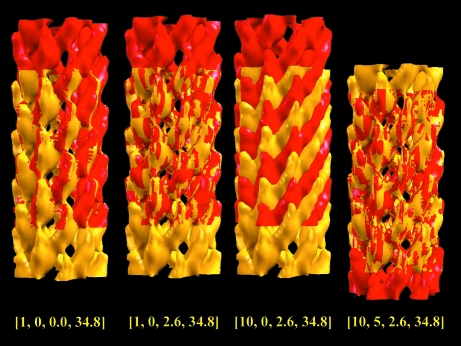
The procedure of serial density-map superimpositions illustrates the determination of the 1-D helical symmetry from a helical EM structure. The bateriophage fd coat protein (EMD-1240) is used here for demonstration. The original EM structure is displayed as a yellow isosurface and the symmetry-transformed density map is shown as a red isosurface. A first superimposition, denoted by the 1-D helical symmetry operation [1, 0, 0.0, 34.8], gives the result of a 34.8 Å translation only. Following an additional rotation of 2.6° denoted by [1, 0, 2.6, 34.8], the outcome of perfect superposition determines the [ϕ, δ] of 1-D helical symmetry for the set of ten-start primary protofilaments. Applying a tenfold rotation [10, 0, 2.6, 34.8] then gives the third superimposition. Finally, an extra translation defined by *n*_2_ = 5 completes the determination of 1-D helical symmetry [10, 5, 2.6, 34.8] for the EMD-1240 structure.

To illustrate the procedure of 1-D helical symmetry determination, four superimpositions between the original EM (EMD-1240; bateriophage fd coat protein B; Wang *et al.*, 2006 ▶) and transformed density maps relating to the four stages are given in Fig. 7 ▶. The first two superimpositions illustrate the determination of [ϕ, δ] for the assigned primary protofilament. The last two superimpositions illustrate the determination of the secondary protofilament *n*_2_, giving the 1-D helical symmetry [10, 5, 2.6, 34.8]. The 1-D helical symmetry for each helical EM map deposited in the EMDB has been determined with the graphics tool *PNAS*. Some of the results are listed in Table 1 ▶ and a complete list is reported on the webpage http://protein3d.ncifcrf.gov/helicalSymmetry/table1.html. To highlight the importance of a comprehensive helical scheme, Table 2 ▶ provides a comparison between the reported helical symmetries determined in the EM reconstruction and the new helical symmetries for six polymorphic helical structures of the microtubule. The results clearly show that the inherent structural characteristics of the microtubule obtained by the new helical scheme can directly discover polymorphic ensembles. The very similar surface lattice within different helical symmetries implies very similar subunit–subunit interactions which the microtubule uses to assemble into divergent helical organizations.

## 4. Discussion

### 4.1. Will the guidelines give a unique [*n*_1_, *n*_2_, ϕ, δ] helical system?

**Figure 8:**
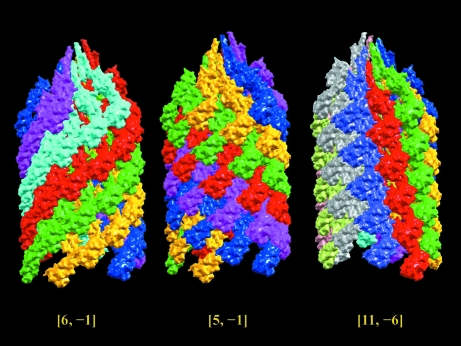
1-D helical symmetry determination for a complicated helical structure. This example illustrates that the guideline defined for 1-D helical symmetry determination is capable of giving a unique symmetry assignment [*n*_1_, *n*_2_, ϕ, δ] to an intricate helical structure. The asymmetric subunit of the bacterial flagellar hook (Fujii *et al.*, 2009 ▶; PDB entry 3a69; EMD-1647) contains three protein domains spanning the inner, middle and outer layers of the helical structure. Based on individual domains, the best protofilament in each domain forms a set of six-start, five-start and 11-start helices, respectively, from the outer to the inner layer of the helical structure. The pictorial helical descriptions for three different symmetry assignments are given under [6, −1], [5, −1] and [11, −6] symmetry. The guideline prefers the [11, −6] symmetry assignment simply because the 11-start helix has a twist angle closest to zero and the six-start helix is the protofilament with the largest number of contacts between the asymmetric units along the protofilament.

A helical description using four parameters [*n*_1_, *n*_2_, ϕ, δ], determined according to the augmented 1-D helical symmetry guidelines (in §[Sec sec2]2) provides the helical signature of the structure. This is because the two sets of defined helices, the *n*_1_-start and the *n*_2_-start, correspond to the two sets of protofilaments. However, will the guidelines also always give a unique [*n*_1_, *n*_2_] combination for a given helical structure? The answer is yes, as illustrated by the example below. The docked atomic model of the bacterial flagellar hook (Fujii *et al.*, 2009 ▶; PDB entry 3a69) contains an asymmetric subunit with three protein domains spanning the inner, middle and outer layers of the helical cryo-EM map. In terms of individual protein domains, the best protofilament of each domain yields an 11-start, five-start and six-start helix, respectively, from the inner to the outer layers. Even though different helical descriptions of different layers are observed, the guidelines still give an unambiguous helical symmetry of [11, −6, −7.31, 45.32] for this structure. The assignment is based on two clear elements in the structural data: the 11-start helix (the third protofilament in the protein) has a twist angle closest to zero and the six-start helix is the first protofilament. In Fig. 8 ▶, each color presents an assigned helix and the assembly illustrates six, five and 11 helices, respectively, for the [6, −1], [5, −1] and [11, −6] symmetries.

**Figure 9:**
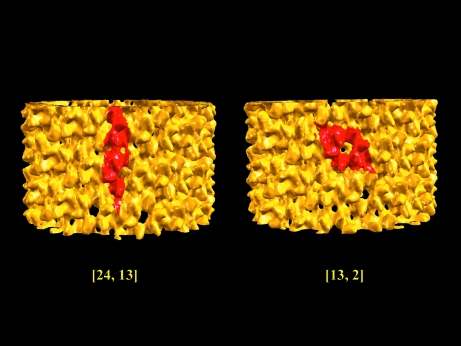
Different surface lattices with different helical symmetry assignment. The span of the surface lattice of the tubular structure of HIV-1 capsid protein (EMD-5136) is highlighted in red for two helical symmetries of [24, 13] and [13, 2].

Not all helical structures have unambiguous primary protofilaments, especially when the growth mechanism does not follow a helical path. The tubular structure of the HIV-1 capsid protein (CA; Byeon *et al.*, 2009 ▶) is such an example. In solution, CA forms a dimer *via* the association of its C-­terminal domain (CTD). The cryo-EM tubular structure (EMD-5136) reveals that the basic unit is a trimer of CA dimers with a pseudo-threefold at the CTD–CTD interfaces and the CA dimer is shared between two trimers. Following our guideline, we obtain a helical symmetry of [24, 13, 7.39, 165.78] for the CA tubular structure. The unit cell depicted by the [24,13] symmetry does not correspond to the observed CA hexamer; however, after applying the symmetry-manipulation rules (*n*_1_ = *n*_1_ − *n*_2_, [*n*_1_, *n*_2_] swapping and *n*_2_ = *n*_2_ + *n*_1_) the new helical symmetry of [13, 2, −11.00, 89.80] gives the cell dimensions of the hexamer. The surface lattices for both helical symmetries are highlighted in red in Fig. 9 ▶. The fact that the assigned asymmetric units in both helical symmetries ([24, 13] and [13, 2]) do not correspond to the assembly unit implies that the path of a trimer of CA dimers is not helical. In this case, our guidelines will fail to offer an unambiguous helical specification.

The guidelines have two limitations in fulfilling the aim that every helical structure would have a unique [*n*_1_, *n*_2_, ϕ, δ] helical symmetry. The first arises when the primary protofilament is ambiguous, as discussed above, and the second is encountered when there is a continuous helical density along a protofilament in the cryo-EM structure rather than a clear boundary between asymmetric units. Under such circumstances, for a determined [*n*_1_, *n*_2_] symmetry the helical structure can be described by an infinite number of [ϕ, δ] pairs, which are always related by a constant. The helical structure of the tubular Aβ_1–42_ amyloid with a hollow core (Miller *et al.*, 2010 ▶; Zhang *et al.*, 2009 ▶) is an example of this limitation. The cryo-EM structure gives a [2, 0] (or [2, 1]) helical symmetry and the two helical parameters [ϕ, δ] = [−3.75*c*, 4.8*c*], where *c* is a constant.

### 4.2. Relevance to experimental diffraction patterns

The diffraction patterns of helical structures consist of a series of layer-lines. Assuming that the layer-lines do not overlap, each layer-line is the result of diffraction by a set of *n*-­start helices. The position of the peak with the maximum diffraction intensity in each layer-line can be indexed to correspond to a node in the helical net. The relationship between the 1-D helical system [*n*_1_, *n*_2_] symmetry and the diffraction pattern is detailed in Figs. 4 ▶(*a*) and 4 ▶(*b*). The two figures illustrate the different assignments of *n*_10_ and *n*_01_, which give different helical symmetries, [3, 1] and [3, −2], for the same structure that has a simple helical symmetry of *t* = 4 and *u* = 13. The assignment of *n*_10_ with the first peak close to the equator (*n*) of the diffraction pattern is consistent with the guideline for selecting the *n*_1_-start helices with a twist angle closest to zero. The assignment of *n*_01_ to the position of the diffraction which is close to the origin is also likely to constitute a main protofilament of the given helical structure.

### 4.3. The minimal number of helices needed for a complete helical structure description

From the rotohelical transformation, we learnt that a single (one-start) helix description is not always sufficient to generate the entire helical structure from given asymmetric units. The helix may need to be related by a *C_n_* rotational symmetry, which implies that a minimum of *n* helices are required to cover the entire helical assembly. Given an [*n*_1_, *n*_2_] symmetry, there are *n*_1_ or *n*_2_ assigned helices for the entire helical description. To determine the minimal number of helices for complete structural description (or to be correlated with the rotohelical transformation), the [*n*_1_, *n*_2_] symmetry is reduced to an equivalent symmetry with *n*_2_ = 1 or 0. In the case of a reduced [*n*_1_, 0] symmetry, the new *n*_1_ is the minimal number of helices.

It is straightforward to deduce the minimal number of helices for a given [*n*_1_, *n*_2_] helical symmetry. If the numbers *n*_1_ and *n*_2_ do not have a common factor, the symmetry can always be reduced to a one-start helix description by using a combination of the swap and the equivalence rules of *n* = *h* *n*_10_ − *k* *n*_01_ as described in §[Sec sec2]2. For example, [7, 3] can be reduced to [1, 3] with *h* = 0, *k* = 2. On the other hand, the largest common factor between *n*_1_ and *n*_2_ is the minimal number of helices for a complete helical structure description. For example, [8, 4] can be reduced to [4, 0] with the number 4, the largest common factor of 4 and 8.

### 4.4. A new description of helical symmetry

Despite the fact that so many helical structures have been determined, a universal formulation for representing helical symmetry is still lacking. The absence of agreement in the community has been attributed to three main reasons. The first apparent reason is a consequence of the fact that helical symmetries have been formulated in distinct ways to fulfill a particular requirement or convenience in different structure-determination methods. The diversified helical representations can be classified into two commonly adopted helical schemes named the 1-D and 2-D helical systems. In this study, the two helical schemes were unified into a single helical specification by two constants [*n*_1_, *n*_2_] and we have shown that the two systems are interchangeable and complementary to each other. Because of the simplicity of using one less parameter and the lack of involvement of the axial radius, we suggest using the augmented 1-D helical system with four parameters [*n*_1_, *n*_2_, twist, rise] for representing a helical structure.

The second hurdle for defining a helical description is that a helical structure can be pictured in many ways, *i.e.* in many [*n*_1_, *n*_2_] combinations as two (*n*_1_-start and *n*_2_-start) sets of helices. However, in principle, the generalized guidelines for describing a helical symmetry are expected to give a unique [*n*_1_, *n*_2_] specification that reflects the characteristics of the structure, although in a limited number of cases a unique specification is impossible.

The fact that no standard helical symmetry has been accepted so far can be attributed to the last obstacle: a com­plete coverage of helical description includes the capability of handling helical discontinuity (a seam). However, building an entire helical construct with a seam from given asymmetric units requires no additional modification in our formulation of helical transformation. Instead, a helical structure with a seam is simply reflected in the value of *n*_2_. By definition, the helical discontinuity indicates that *n*_2_ is no longer an integer but a rational number.

### 4.5. Presentation of a structure with a helical discontinuity

An implicit requirement of the 2-D helical system (§[Sec sec2.1]2.1) is that in a seamless helical arrangement [*n*_1_, *n*_2_] must be specified by integer numbers. By treating two consecutive asymmetric subunits in the primary protofilament as a new single asymmetric subunit, the new augmented 1-D helical symmetry becomes [*n*_1_, *n*_2_/2, 2ϕ, 2δ], which is equivalent to the original helical symmetry [*n*_1_, *n*_2_, ϕ, δ] except that the asymmetric units are doubled in size. When the tubulin subunit is treated not as a dimer of αβ subunits but as a single subunit by ignoring the small difference between the α and β subunits (Sui & Downing, 2010 ▶), we do not encounter the microtubule seam problem. However, when treating the αβ dimer as an asymmetric subunit in the new 1-D helical symmetry, helical structures with an odd number for the *n*_2_ symmetry (in single subunit representation) create a seam with a new rational *n*_2_.

The microtubule EM structure (Cochran *et al.*, 2009 ▶; EMD-5038) presents such a helical discontinuity when treating the dimer of αβ subunits as the asymmetric unit. The augmented 1-D helical symmetry in four parameters [13, 3/2, 0.0, 80.0] is sufficient to generate the entire helical structure with a seam, based on the helical transformation matrix summarized in Fig. 2 ▶. Owing to the helical discontinuity, the repetitive asymmetric unit is no longer an identical unit. Instead, a complete round of *n*_1_ subunits (13 dimers in the microtubule case) now constitutes the identical unit in the helical structure with a seam. Therefore, the subunit coordination index [*m*_1_, *m*_2_] can no longer have an index with *m*_1_ ≥ *n*_1_ when applying the helical transformation to generate the repetitive subunits for a helical structure with a seam.

A seam in a helical structure can be classified visually with respect to its helical axis into a strictly vertical seam or a seam that wraps around the helix. The microtubule case above is an example of a vertical seam. Under the restriction that only a rational *n*_2_ and integer *n*_1_ > |*n*_2_| are allowed in the augmented 1-­D helical representation, the corresponding helical structure always produces a vertical seam and the handedness of the seam is determined by the sign of *n*_2_, with positive indicating a left-handed seam and negative a right-handed seam. In con­trast, a seam described by a rational *n*_1_ > |*n*_2_| and integer *n*_2_ should correspond to the type of seam that wraps around the helix.

### 4.6. Application to polymorphic structural assemblies

Both the 1-D and 2-D helical systems are designed to create helical assemblies from asymmetric subunits with specified helical parameters. The conformational heterogeneity of molecular assemblies is known to set limits on solving cryo-EM structures at high resolution. Polymorphism is particularly problematic in the determination of structures with helical symmetry since even a slight deviation in the interactions between two asymmetric subunits will create distinct structures with different symmetries. We have seen such an example in Table 2 ▶ for the microtubule structure. The question is can all such polymorphic structures be generated based on a single helical structure which is given in an atomic model or an EM map? The answer is yes, because the interactions between the asymmetric subunits are preserved in the definition of the 2-D helical system. Thus, to create distinct polymorphic structures with almost the same subunit–subunit interactions we only need to change the specific [*n*_1_, *n*_2_] helical symmetry.

## 5. Conclusions

In this paper, we give two helical formulations (augmented 1-­D and 2-D) to describe a helical structure. Unlike the rotohelical transformation (1-D formulation) with a helical plus an additional rotational operation, a new augmented 1-D formulation with two consecutive helical operations enables unification with the widely adopted 2-D formulation, giving a common helical symmetry descriptor with two integers [*n*_1_, *n*_2_]. The new formulation requires only four parameters [*n*_1_, *n*_2_, twist, rise] for the augmented 1-D helical system and five parameters [*n*_1_, *n*_2_, *a*, *b*, γ] for a 2-D helical system to generate the entire structural assembly from given asymmetric units. We propose using the augmented 1-D helical system with four parameters to describe a helical structure owing to its simplicity and independence from the helical radius compared with the 2-D helical system.

In terms of a helical net representation, a helical structure with an [*n*_1_, *n*_2_] symmetry indicates that its organization is specified by two sets of helices (*n*_1_-start and *n*_2_-start). Because many different [*n*_1_, *n*_2_] combinations exist for the same structure, we suggest general guidelines for selecting a unique [*n*_1_, *n*_2_] symmetry which reflects the structural characteristics of a given helical structure. We provide a computational graphics tool for this purpose which can be used for any helical structure determined by X-ray fibre diffraction or EM imaging.

While there are multiple ways to construct equations that generate the same helical structure, an [*n*_1_, *n*_2_, twist, rise] description provides the following advantages: firstly, it provides full helical coverage, including a helical discontinuity (seam) which is indicated by a rational *n*_2_; secondly, it reflects the structural characteristics of the assembly (formation mechanism) directly by four helical parameters; that is, similar structures give similar parameters; thirdly, the unnecessary error in reproducing the entire helical structures, such as editing wrong transformation matrices in the PDB or in the deposited EM parameters in the EMDB, will be prevented; and lastly, the new helical symmetry is expected to be useful for maintaining a pre-determined helical symmetry in structural refinement as well as for the generation of all ‘meaningful’ polymorphic structural assemblies from a given helical atomic model or EM density map.

## Figures and Tables

**Table 1:** Helical parameters for helical structures solved by X-ray fibre diffraction and EM imaging The first and second columns provide the PDB code and molecular name of the helical structure. The third column indicates whether the helical assembly was solved by X-ray fibre diffraction (XFD) or cryo-EM imaging (CryoEM). If the structure was solved by cryo-EM imaging, the PDB file records atomic models which have been docked into the corresponding EM map. If the EM map was deposited in EMDB, the entry gives its EMDB ID code. The next three columns (*C_n_*, ϕ, δ) report the helical symmetry if specified in the PDB or EMDB. The following two columns give the unified helical symmetry of [*n*_1_, *n*_2_] by following the symmetry-determination guideline (provided in the text). Next are the newly determined helical parameters [ϕ, δ] of the 1-D helical system and the lattice constants (*a*, *b*, γ) of the 2-D helical system.

PDB code	Molecule	XFD/cryoEM	*C_n_*	Twist ϕ (PDB) (°)	Rise δ (PDB) (Å)	*n*_1_	*n*_2_	Twist ϕ (1-D) (°)	Rise δ (1-D) (Å)	*a*	*b*	γ
1ifd	Inovirus	XFD	*C*_5_	−33.23	16.00	10	−5	5.54	32.00	21.16	32.06	37.35
1hgv	PH75 inovirus	XFD	*C*_1_	66.67	2.90	11	−6	13.33	31.90	23.05	32.29	32.01
1cgm	Mosaic virus	XFD	*C*_1_	22.04	1.44	16	−1	−7.34	23.11	22.59	24.31	104.34
2zwh	F-actin model	XFD	*C*_1_	−166.40	27.59	2	1	27.20	55.18	69.06	56.14	77.07
3a5x	L-type flagellar	CryoEM	*C*_1_	65.30	4.79	11	5	−1.70	52.65	42.96	52.68	125.82
1mwk	ParM filament open	EMD-5128	*C*_1_	165.20	24.30	2	−1	−29.50	48.60	62.30	49.67	78.94
1mwk	ParM filament closed	EMD-5129	*C*_1_	165.00	24.20	2	−1	−30.10	48.60	62.75	49.73	79.47
2hi5	Bacteriophage	EMD-1240	*C*_5_	−34.62	17.40	10	−5	2.60	34.80	22.50	34.82	138.88
3b5u	Acrosomal actin	EMD-1088	*C*_1_	−164.90	27.75	2	1	23.34	54.60	67.74	55.27	75.18
3dik	HIV-1 CA	EMD-5136				13	2	−11.00	89.80	96.64	96.68	119.96
3a69	Flagellar hook	EMD-1647	*C*_1_	64.79	4.12	11	−6	−7.31	45.32	38.37	45.93	52.25
3dco	Microtubule	EMD-5038				13	3/2	0.00	80.00	51.94	80.00	100.24

**Table 2:** Comparison of helical parameters between the reported symmetries determined in the EM reconstruction and the new helical symmetries for six polymorphic microtubule structures See Table 1 ▶ for column name description. The last column gives the helical radius where the 2-D lattice was defined.

CryoEM	*C_n_*	Twist ϕ	Rise δ	*n*_1_	*n*_2_	Twist ϕ (1-D)	Rise δ (1-D)	*a*	*b*	γ	Radius (COD)
EMD-5191	*C*_1_	−32.47	11.08	11	3	0.95	40.6	52.58	40.63	100.03	90.71
EMD-5192	*C*_1_	−29.88	10.16	12	3	0.50	40.6	52.35	40.61	99.97	98.49
EMD-5193	*C*_1_	−27.69	9.39	13	3	0.00	40.6	52.59	40.60	100.26	107.07
EMD-5194	*C*_1_	−25.77	8.72	14	3	−0.25	40.6	51.92	40.60	100.35	113.81
EMD-5195	*C*_1_	−23.83	10.81	15	4	0.65	40.6	51.00	40.62	100.34	119.85
EMD-5196	*C*_1_	−22.40	10.18	16	4	0.40	40.6	51.12	40.61	100.19	128.16
